# Management of sellar and parasellar tumors becoming symptomatic during pregnancy: a practical algorithm based on multi-center experience and systematic literature review

**DOI:** 10.1007/s11102-020-01107-2

**Published:** 2020-11-17

**Authors:** Matteo Zoli, Federica Guaraldi, Cesare Zoia, Emanuele La Corte, Sofia Asioli, Daniele Bongetta, Arianna Rustici, Diego Mazzatenta

**Affiliations:** 1 Pituitary Unit, IRCCS Istituto Delle Scienze Neurologiche di Bologna, Via Altura, 3, 40139 Bologna, Italy; 2 Department of Biomedical and Neuromotor Sciences (DIBINEM), University of Bologna, Bologna, Italy; 3 Neurosurgery Unit, Fondazione IRCCS Policlinico San Matteo, Pavia, Italy; 4 Unit of Anatomic Pathology ‘M. Malpighi’, Bellaria Hospital, Azienda USL Bologna, Bologna, Italy; 5 Neurosurgery Unit, ASST Fatebenefratelli Sacco, Milano, Italy; 6 Department of Experimental, Diagnostic and Specialty Medicine (DIMES), University of Bologna, Bologna, Italy

**Keywords:** Pituitary tumors, Pituitary adenoma, Meningioma, Pregnancy, Management, Outcome

## Abstract

**Introduction:**

Sellar/parasellar tumors (SPTs) very rarely become symptomatic during pregnancy. No specific guidelines exist for their management, that is extremely challenging as mother and fetus health can be jeopardized.

**Materials and methods:**

Data of patients with SPTs becoming symptomatic during pregnancy treated at two Italian referral Centers were retrospectively collected. Systematic literature review was also performed.

**Results:**

Our series consisted of 6 cases, 3 meningiomas, 1 ACTH-secreting adenoma, 1 pituicytoma and 1 craniopharyngioma. Mean age at presentation was 33.6 ± 6.0 years. Five patients complained of visual disturbances, associated with headache in one case, that occurred between gestation week (GW) 22 and 34. In 5 cases, pregnancy was uneventful with the delivery of a healthy baby between GW 33 and 35, followed by endoscopic surgical tumor exeresis (n = 4) or proton bean therapy (n = 1). Another patient presented with stigmata typical of Cushing’s syndrome and rapidly worsening pre-eclampsia, that required pregnancy interruption and adenomectomy. Based on personal and literature cases, a practical algorithm was proposed to help clinicians dealing with these patients.

**Conclusions:**

SPTs becoming symptomatic in pregnancy deserve careful monitoring and multidisciplinary management. Overall, wait-and-see approach is suggested, reserving surgery to patients with rapidly progressive/life-threatening situations, significant risk of permanent neurological impairment or malignant lesions.

## Introduction

Sellar/parasellar tumors (SPTs) can enlarge during pregnancy as a consequence of multiple mechanisms [1–5]. Pituitary volume physiologically enlarges because of estrogen-stimulated hypertrophy and hyperplasia of lactotroph cells, the last supporting the greater propensity of prolactinomas and pluri-hormonal adenomas to increase in size during pregnancy. In meningiomas, rise of progesterone levels, acting via specific receptors, may further contribute to increase tumor volume and, thus the pressure on nearby neural structures [1–3, 5].

In the great majority of the patients with SPTs, pregnancy is uneventful as lesions are typically small and the overall size increase during gestation is not clinically significant [1–3]. Rarely, tumor enlargement can determine mass effect on adjacent anatomic structures, with consequent neurological and visual symptoms [1, 3–25]. Additionally, hormone secretion can be impaired by tumor compression of the pituitary gland and deviation of the stalk, or by the presence of a secreting adenoma, with detrimental effects on fetal development and pregnancy outcome [26]. Finally, the increase of adenoma size is associated with a higher risk of pituitary apoplexy and of consequent acute hypopituitarism, typically occurring in the last weeks of pregnancy and at delivery [1–25].

Moreover, the diagnosis of a SPT in pregnancy is particularly challenging. Indeed, physiological modification in size and function, secondary to pituitary adaption to placental hormonal secretion, complicates the interpretation of biochemical and imaging test [1–3, 5]. Furthermore, CT-scan should be avoided because of ionizing radiations, while the execution of MRI and the administration of gadolinium contrast medium requires precautions, especially in the first trimester [1–3]. Therefore, visual field examination, be repeated at regular intervals, is reported as an useful investigation for SPTs manifesting with visual disturbances in pregnancy, to monitor the further tumor growth [27].

Once diagnosis is established, each case has to be carefully discussed by a multidisciplinary dedicated team made of neuroendocrinologists, anesthesiologists, pituitary neurosurgeons, gynecologists and obstetricians. Indeed, their management has to be patient-tailored with the aim is to restore the mother clinical conditions, while preserving fetus health [1, 5, 11, 21].

Goal of our study is to present our clinical experience and the results of a systematic literature review on the management of SPTs becoming symptomatic during pregnancy. We were, also, aimed to propose a practical algorithm to help clinicians dealing with this condition.

## Materials and methods

Data of patients with SPTs consecutively referred to two Italian referral Centers (IRCCS Istituto delle Scienze Neurologiche di Bologna, Bologna, Italy, and Fondazione IRCCS Policlinico San Matteo, Pavia, Italy) from 2000 to 2019 were retrospectively reviewed to identify cases becoming symptomatic during pregnancy. Clinical, biochemical and radiological data of interest at first evaluation and at follow-up were retrieved from paper and electronic medical records.

At presentation, patients underwent neurological and ophthalmological evaluation—including visual field and visual acuity measurement -, and endocrinological evaluation with biochemical assessment of basal anterior (TSH, free T4, free T3, ACTH, cortisol, prolactin, GH, IGF-I) and posterior pituitary function (serum values of sodium and potassium and plasmatic and urinary osmolarity). An MRI without contrast medium was also performed to confirm the presence and characterize the SPT in all cases. The management of every case was discussed collegially.

Tumor specimens were reviewed and classified according to the WHO classification of 2016 of central nervous system and endocrine organs tumors, respectively [28, 29]. Samples were fixed in 10% formalin and embedded in paraffin. Tissue was cut into sections of 4-μm thickness and stained with hematoxylin and eosin. Immunohistochemical staining was performed by an automatic system (Ventana Benchmark, Ventana Medical Systems, Illkirch, France), using avidin–biotin labeling and diaminobenzidine as detection reagent. Immunohistochemistry was performed using the BenchMark ULTRA Slide Staining System (Roche Diagnostics, Indianapolis, IN, USA). Primary antibodies varied according to the diagnostic suspect [28]. Specifically, anti-chromogranin A (Dako Agilent, Santa Clara, CA, USA; monoclonal Dak-A3; dilution 1:200), pancytokeratin (Novocastra Newcastle, UK; monoclonal AE1 and AE3; dilution 1:50), FSH (Cell Marque, Darmstadt, Germany; monoclonal 83/122A8 antibody; dilution 1:2000), LH (Cell Marque, Darmstadt, Germany; monoclonal 3LH5B6Y antibody; dilution 1:100), TSH (Cell Marque, Darmstadt, Germany; monoclonal 5404 antibody; dilution 1:50), PRL (Biogenex, Fremont, CA, US; monoclonal BGX031A; dilution 1:50), ACTH (Dako Agilent, Santa Clara, CA, US; monoclonal, clone 02A3; dilution 1:1000), GH (Cell Marque, Darmstadt, Germany; monoclonal 54/92A2; dilution 1:50), Steroidogenic Factor 1 (Abcam, Cambridge, UK; monoclonal EPR19744; 1:150), PIT1 (Novus Biologicals, Abingdon Oxon, UK; polyclonal; dilution 1:100), TPIT (Atlas Antibodies, Stockholm, Sweden; monoclonal CL6251; 1:300) were used to diagnose and classify pituitary adenomas [28–30]. Anti-EMA (Novocastra, Newcastle, UK; monoclonal GP1.4; dilution 1:500) and anti- progesterone receptor (Ventana, Oro Valley, AZ, US; 1E2 clone; pre-diluted) were used for the diagnosis of meningiomas; anti-vimentin (Dako Agilent, Santa Clara, CA, US; monoclonal SRL-33; dilution 1:200), anti-S-100 (Dako Agilent, Santa Clara, CA, US; polyclonal; dilution 1:1000), GFAP (Dako Agilent, monoclonal 6F2; dilution 1:1000), TTF-1 (Novocastra, Newcastle, UK; monoclonal SPT24; dilution 1:50; and Dako Agilent, Santa Clara, CA, US; 8G7G3/1; dilution 1:100) and EMA (Novocastra, Newcastle, UK; monoclonal GP1.4; dilution 1:500) for pituicytoma; anti-b-catenin (Ventana/ Roche, monoclonal clone 14; dilution 1:1) for the identification of craniopharyngiomas and anti-BRAFV600E (Ventana, VE1 clone; prediluted) for differential diagnosis between adamantinomatous and papillary subtype [28, 29].

Exeresis was considered radical in the absence of tumor remnants at the MRI; subtotal if the remnant was < 20%, partial if 20–50%, and incomplete if > 50% of the initial mass. Endocrinological, ophthalmological and neurological evaluations, as well as biochemical and functional tests, were repeated 1, 3, 6 and 12 months after surgery, then annually. MRI was repeated 3 months after surgery, then every 6–12 months, depending on the clinical, radiological and histological features, following international guidelines.

## Literature review

### Search strategy

**Fig. 1:**
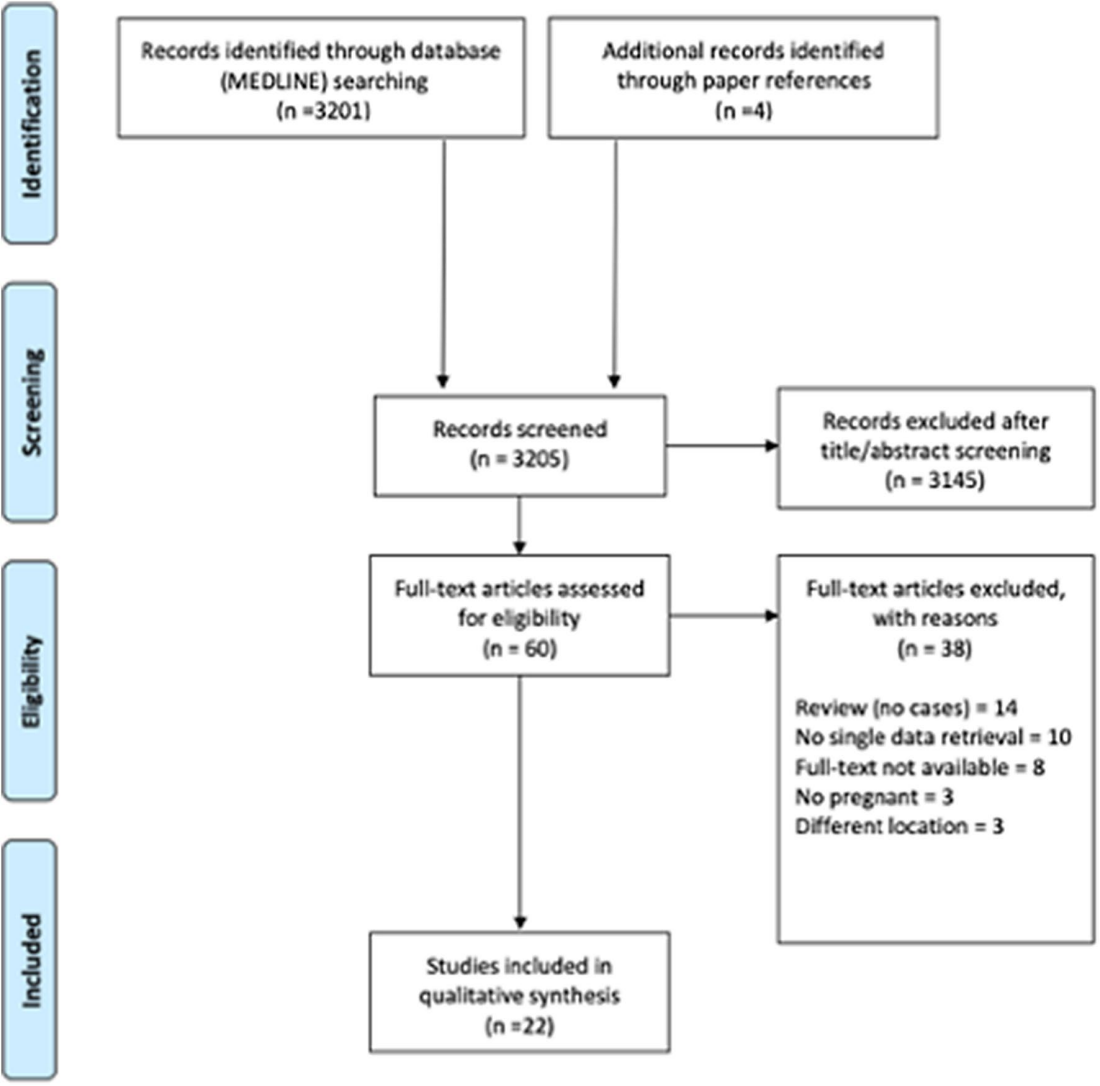
Flow-chart applied to the retrieval and selection of studies included in literature review

Systematic literature review was performed in accordance with the Preferred Reporting Items for Systematic Reviews and Meta‐analyses (PRISMA) guidelines (Fig. 1). MEDLINE database was queried using keywords and MeSH terms in different combinations using the Boolean operators “AND” or “OR”, and database-related filters to maximize the chance to identify articles focusing on surgical management of patients with SPTs developing symptoms during pregnancy. The string ((‘brain tumor’ OR ‘brain tumour’ OR ‘pituitary tumor’ OR ‘pituitary tumour’ OR ‘sellar lesion’ OR ‘pituitary disease’) AND pregnancy) was entered. Search was limited to original studies written in English, performed in human subjects and published after 2000 (in the same time frame of our case series). After duplicate removal, articles were screened on the basis of the title and the abstract; for those deemed appropriate, the full text was obtained and reviewed, and data of interest were extracted. Reference list of selected articles was examined to identify other potentially relevant studies.

### Selection criteria

Only original studies reporting patients with SPTs developing symptoms during pregnancy with clinical, radiological and histological data of interest were included. Articles were excluded if lesions did not primarily involve the sellar/parasellar region, or the disease manifested after delivery. Studies including different surgical procedures and/or patient populations were included only if sufficient data about the management of SPT in each patient could be obtained.

### Data analysis

Personal and literature data were tabled and analyzed using Microsoft Excel 2019 (Microsoft Corp, Redmond, WA). Collected variables included patient age, gestational week and symptoms at diagnosis; gestational week and type of delivery; pregnancy outcome; treatment type, timing (with respect to pregnancy) and outcome. Data were expressed as mean ± SD.

## Results

The series included 6 cases: 3 meningiomas, 1 craniopharyngioma, 1 ACTH-secreting adenoma and 1 pituicytoma, managed at the Pituitary Unit of the IRCCS Istituto delle Scienze Neurologiche di Bologna, and at the Neurosurgical Department of the Fondazione IRCCS Policlinico San Matteo, Pavia (Italy), since 2000 to 2019. In the same period, other 14 pregnant patients affected by SPTs (or diagnosed before pregnancy and untreated with medical or wait and see approach or treated but with MRI-detectable remnant at last follow-up performed before pregnancy) were managed at our Centers. Specifically, 11 patients were affected by pituitary adenomas—6 prolactin-, 2 GH- and 1 ACTH-secreting, and 2 nonfunctioning -, 2 by meningiomas and 1 by craniopharyngioma.

At presentation, patient age was 33.6 ± 6.0 years and gestation week was 29.8 ± 4.7. Presenting symptoms were visual disturbances (n = 5), diplopia (n = 1), headache (n = 1) and features suggestive for chronic hypercortisolism (n = 1). Two cases (#3 and #5) had been previously reported [31, 32]. Significant patient data at presentation are reported in Table 1.

**Table 1:** Main patient clinical features at symptom onset and tumor histotype

Case #	Age	Gestational week	Visual symptoms	Neurological symptoms	Pituitary function	Tumor histotype
1	35	34	BTH	Headache	Intact	Pituicytoma
2	26	22	None	None	Hypercortisolism	ACTH-secreting adenoma
3 [32]	43	32	BTH; VAD	None	Intact	Meningioma
4	37	27	quadrantopia; VAD	None	Intact	Meningioma
5 [31]	32	30	BTH; VAD	None	Partial hypopituitarism	Craniopharyngioma
6	29	34	None	Diplopia	Partial hypopituitarism	Meningioma

*BTH* bitemporal hemianopia, *VAD* visual acuity deficit

Mother and fetus vital parameters were strictly monitored up to delivery (n = 5). Pregnancy was uncomplicated and the newborns were healthy. A patient with ACTH secreting adenoma (case #2) required pregnancy interruption at 24 weeks of gestation.

Five patients underwent endoscopic endonasal surgery after delivery because of symptoms persistence. Surgery was uneventful. Tumor exeresis was radical in 4 (80%) cases, while in one with hemorrhagic craniopharyngioma, the first surgery allowed the decompression of the optic nerve and chiasm, and radical resection was achieved by second surgery, performed two years later (Table 2). A patient with cavernous sinus meningioma had spontaneous regression of diplopia after delivery and underwent proton-beam therapy (Table 2).

**Table 2:** Pregnancy outcome, post-delivery visual, neurological and endocrinological function, treatment type and results

Case #	Pregnancy outcome	Gestational age at delivery/abortion	Type of delivery	Post delivery function			Post-delivery	
				Visual	Neurological	Pituitary	Tumor treatment	Outcome
1	DHB	34	cs	n	n	i	EES	RR
2	Abortion	24	–	i	i	i	EES	RR
3 [32]	DHB	34	cs	n	i	i	EES	RR
4	DHB	34	cs	n	i	i	EES	RR
5 [31]	DHB	33	cs	n	i	panhypopituitarism	EES	SR
6	DHB	35	cs	i	n	i	Proton therapy	Stability

*cs* cesarian section, *DHB* delivery of a healthy baby, *EEA* endoscopic endonasal approach, *I* intact, *n* normalization, *RR* radical resection, *SR* subtotal resection

Mean follow up was 88.6 ± 28.8 months. At last evaluation, patients treated with surgery were free from disease, while the one treated with hadrotherapy had a stable tumor remnant (Table 2).

## Case illustration

### Case 1

A 35-year-old woman at 30 weeks of gestation was referred to neurosurgical attention for the onset of severe headache and progressive visual deficit. Visual field examination revealed an incomplete bitemporal hemianopia. Basal pituitary function was preserved. Fetus vital parameters and development were normal for gestational age. MRI demonstrated the presence of an endo- and suprasellar tumor, compressing the optic chiasm. Wait and see approach with close patient and fetus clinical monitoring and repetition of visual field examination was chosen.

**Fig. 2:**
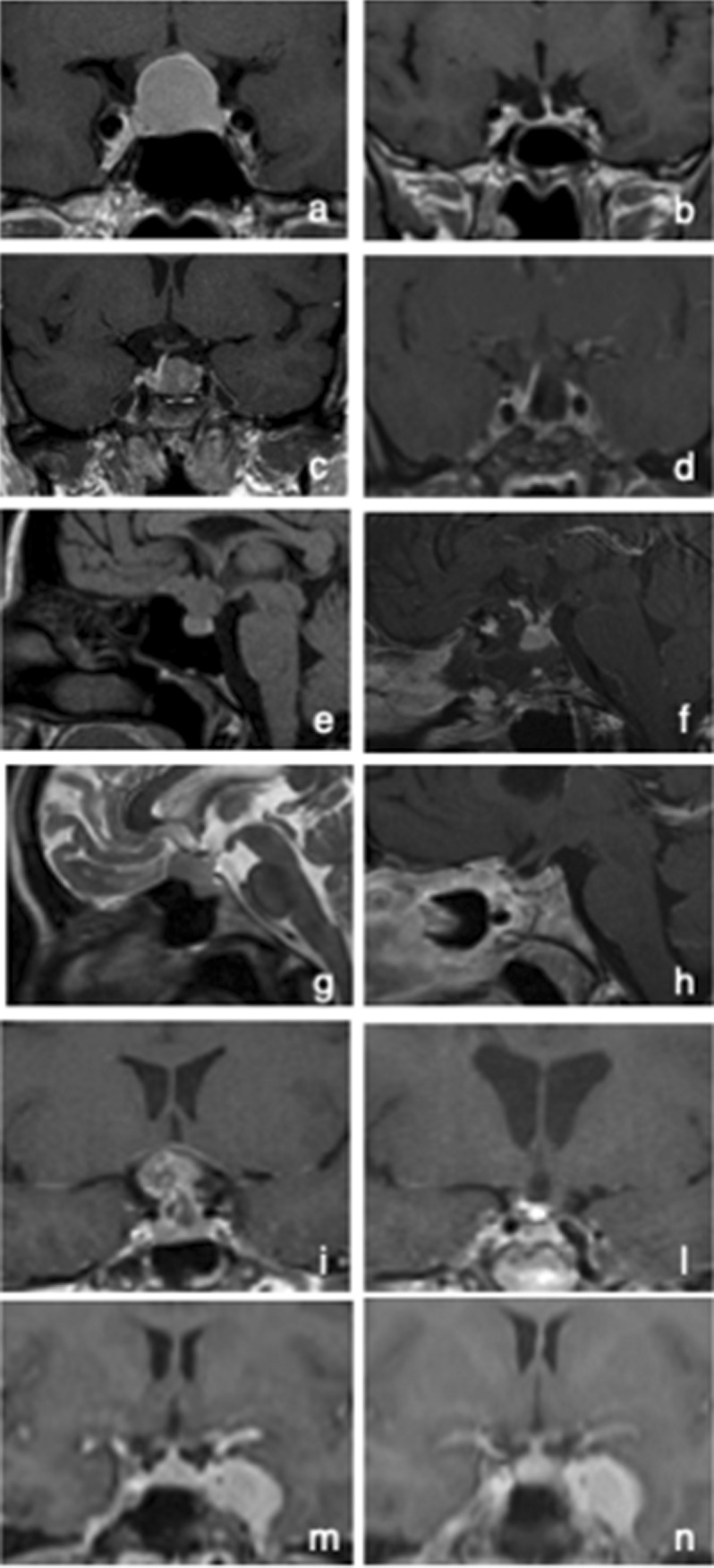
Coronal views of the gadolinium-enhanced T1-weighted pre- and post-operative MRI showing the present of an intrasellar pituicytoma (case #1) and ACTH-secreting macroadenoma (case #2) (**a**, **c**), and their complete exeresis (**b**, **d**). Sagittal view of the gadolinium-enhanced T1-weighted MRI showing a meningioma arising from the tuberculum sellae (**e**), removed through endoscopic endonasal approach, with the preservation of the pituitary gland and stalk (**f**) (case #3). Sagittal views of the pre-operative T2-weighted (**g**), and post-operative T1-weighted gadolinium-enhanced MRI (**h**), showing the complete exeresis of a tuberculum sellae meningioma (case #4). Sagittal views of the gadolinium-enhanced T1-weighted pre- (**i**) and post-operative MRI (**l**), showing a suprasellar craniopharyngioma and its total exeresis after the 2nd endoscopic endonasal surgery, respectively (case #5). Coronal view of the gadolinium-enhanced T1-weighted MRI showing a meningioma occupying the left cavernous sinus (**m**), and the results of hadronic radiation treatment (**n**)

Because of patient persistently complained of anxiety and discomfort associated with visual disturbances, despite the stability of the visual field, after careful gynecological-obstetrician counseling with the evaluations of risks and benefits for the mother and fetus health, elective Caesarian section was performed at 34 weeks of gestation, with no complications. For the potential spontaneous mass reduction, and consequent resolution of compressive effects, after the cessation of pregnancy-associated hormone hyperstimulation, we decided postpone surgery. Since no spontaneous tumor reduction at MRI (Fig. 2a) nor improvement of visual symptoms occurred, the patient underwent elective endoscopic endonasal surgery two weeks after delivery. Tumor resection was radical and uneventful (Fig. 2b). Histopathological examination revealed the presence of bipolar spindled cells arranged in fascicular or storiform pattern, with strong and diffuse staining for vimentin, and nuclear expression of TTF1. GFAP varied from focal to moderate and patcy. EMA staining pattern was patchy and prevalent in cytoplasm. The Ki67 index was low (< 2%). All these features are typical of pituicytoma.

At last follow-up, performed 101 months after surgery, visual field and pituitary function were restored.

### Case 2

A 26-year-old primigravida was referred at 20 weeks of gestation to endocrinological attention for excessive weight gain, facial rubeosis and diffuse increase of body hair from the 16^th^ week of pregnancy. Biochemical evaluations performed in different days demonstrated increased levels of morning serum cortisol (25.7–28.1; n.v. 4–22.3 μg/dl), plasma ACTH (102.3–95.8 pg/ml; n.v. 5–46), 24-h urine free cortisol (UFC, 876–1088 μg/24 h; n.v. 58–403), and midnight salivary cortisol (1.7–2.3; n.v. < 0.2 mg/dl), together with the absence of cortisol suppression at both low- and high dose dexamethasone suppression test.

The clinical and biochemical picture were suggestive for Cushing’s disease, despite the important diagnostic limitations associated with pregnancy [6]. Fetal development and vital signs were in the normal range, therefore a wait and see strategy was chosen. Unfortunately, severe hypertension and diabetes mellitus occurred at 22 weeks of gestation. Because of symptoms worsening and the high risk of mother and fetus complications, after obstetrician consultation, patient opted for pregnancy interruption 2 weeks later.

Subsequent biochemical examination confirmed cortisol hypersecretion (morning serum cortisol 26.1 ng/ml; plasma ACTH 84 pg/ml, 24-h UFC 998 μg/24 h, midnight salivary cortisol 2.1 mg/dl; serum cortisol after low- and high dose dexamethasone suppression test > 2 μg/dL). Gadolinium-enhanced MRI showed an endo-suprasellar pituitary adenoma, that was removed by endoscopic endonasal approach with no complications (Fig. 2c). Histological examination confirmed the presence of an ACTH-secreting adenoma. Biochemical, clinical and radiological follow-up demonstrated disease remission (Fig. 2d). Two years later, the patient had a spontaneous uncomplicated pregnancy.

### Case 3

A 43-year-old woman at 32 weeks of gestation of her second pregnancy presented to neurosurgical attention for bitemporal hemianopia and significant bilateral reduction of visual acuity in the previous 2 weeks. At admission, visual acuity was 1/10 in the left eye and 4/10 in the right eye. MRI without contrast medium demonstrated the presence of a suprasellar meningioma arising from the tuberculum sellae (Fig. 2e). Basal pituitary function was normal. Fetus vital parameters and development were normal for gestational age. For progressive worsening of visual disturbances, dexamethasone was administered to accelerate the fetal lung maturation, and cesarean delivery was performed at 34 weeks of gestation. The patient underwent endoscopic endonasal surgery 13 days after delivery. Histological examination revealed a meningotheliomatous meningioma (WHO grade 1). Post-surgical evaluations demonstrated normal pituitary function, complete tumor resection and recovery of visual acuity (Fig. 2f).

### Case 4

A 37-year-old woman was referred to neurosurgical attention at 27 weeks of gestation for headache and visual disturbances in the previous month. Visual field examination revealed an infero-temporal quadrantopia associated with residual visual acuity of 2–3/10 in left eye. MRI without contrast medium showed a suprasellar meningioma arising from the tuberculum sellae (Fig. 2g). Basal pituitary function was normal. Fetus conditions and development were normal for gestational age. The clinical and ophthalmologic situation remained stable and after obstetrician evaluation, and corticosteroids administration to induce lung maturation, patient underwent caesarian delivery at 34 weeks of gestation. Tumor removal by endoscopic endonasal was performed 15 days after surgery. Visual disturbances completely regressed. MRI demonstrated the radical resection of the tumor, consisting of a meningotheliomatous meningioma (WHO grade 1) (Fig. 2h).

### Case 5

A 32-year-old woman was referred to neurosurgical attention at 30 weeks of gestation for severe bilateral reduction of visual acuity. Visual field examination documented temporal hemianopia in the right eye and an inferior-temporal field cut on the left. MRI showed an extra-axial lesion in the intra- and suprasellar region, isointense in T1-weighted and iso-hyperintense in long-TR sequences (Fig. 2i). Wait and see approach with close mother and fetus monitoring was chosen. In the meanwhile, corticosteroids were started to induce lung maturation and treat central hypocortisolism, diagnosed at hospital admission.

For the worsening in visual acuity and narrowing of visual field, delivery via caesarean section was performed at 33 weeks of gestation. MRI performed with contrast medium on the same day showed a recent intra-tumoral hemorrhage, that was confirmed at surgical inspection. The patient underwent endoscopic transsphenoidal surgery with subtotal tumor resection and decompression of the optic chiasm, with significant improvement at visual field examination. Histological examination revealed an adamantinomatous craniopharyngioma. For the progressive growth of the tumor remnant in the subsequent 2 years, the patient underwent second endoscopic transsphenoidal surgery with radical tumor exeresis (Fig. 2l).

### Case 6

A 29-year-old woman was referred to neurosurgical attention at 34 weeks of gestation for diplopia in all directions of gaze associated with a meningioma of the left cavernous sinus. Basal pituitary function was normal. Fetus development and vital parameters were normal for gestational age. Wait and see approach with close clinical monitoring was chosen. Clinical conditions remained stable. A cesarean section was performed at the 35th week of gestation, after obstetrician indication after careful evaluations of benefits and risks for mother and foetus. The MRI performed with contrast medium confirmed the presence of a meningioma growing in the left cavernous sinus (Fig. 2m). Diplopia spontaneously disappeared in the following weeks. Three months later the patient underwent hadrontherapy (total dose 55.8 Gy in 31 fractions, administered using IMPT—Intensity Modulated Particle Therapy—technique). At last follow-up, performed 32 months later, tumor dimensions were stable with no neurological symptoms, nor endocrinological deficits (Fig. 2n).

## Literature review

Systematic literature review led to the identification of 50 cases of SPT, clinically presenting in pregnancy [1, 3–25]. At diagnosis, patient age was 31.8 ± 5.1 years (range 18–43; data available for 47 patients) and gestational week was 26.0 ± 7.5 (range 5–40; data available for 46 cases). Gestational age at symptom presentation was 24.2 ± 7.3 weeks (range 5–40; data available for 38 cases). Symptoms mainly occurred in the second trimester (57.9%), less frequently in the third (36.8%) and rarely in the first (5.2%) (Table 3). Reduction of visual acuity was the most common symptom (86% of the cases) followed by headache (18%), nausea (8%), retro-orbital pain (8%) and oculomotor nerve palsy (6%); proptosis, ophtalmoplegia, facial numbness, sinus congestion, cognitive impairment and loss of consciousness were sporadically reported. Two patients presented with features suggestive for acromegaly (Table 3).

**Table 3:** Results of literature review: patient features, disease manifestations and pregnancy outcome

Author, year	Age (years)^a^	Tumor type	Gestational age at evaluation (weeks)	Gestational age at symptom onset (weeks)	Presenting symptoms	Pregnancy course/complications	Gestational age at delivery (week)	Mode of delivery
Zhong et al. 2019 [25]	36	TS-M	N/A	18	VD	R	36	cs
	29	NFPA	N/A	14	VD	R	40	vl
	28	Pituitary abscess	N/A	10	VD; H; nausea	R	Abortion	–
	36	TS-M	N/A	22	VD	Pre-eclampsia	38	cs
Jemel et al. 2019 [24]	32	PRL-PA (PAp)	37	20	VD; H	R	37	vl
	35	PRL-PA (PAp)	22	21	VD; H	R	37	vl
	30	NFPA (PAp)	24	24	VD; H	R	38	vl
Bachmeier et al. 2019 [23]	30	PRL-PA	36	N/A	VD; H	R	37	cs
Ennaifer et al. 2018 [22]	43	PRL-PA	36	36	H; eye ptosis	R	40	vl
Karaca et al. 2018 [3]	N/A	GH-PA	11	N/A	Acromegalic features	R	N/A	vl
	N/A	GH-PA (PAp)	11	N/A	Ophtalmoplegia	R	32	cs
Priddy et al. 2018 [21]	25	Petro-clival M	5	5	Arm numbness,; ROP; weakness	R	Abortion	–
	31	Sellar M	29	26	VD; proptosis	R	34	cs
	29	Cavernous sinus M	18	N/A	Diplopia; CN III palsy; ROP	acute symptom worsening at 33 GW	35	vl
	32	Adenoid cystic carcinoma	> 26	N/A	VD; ROP; facial numbness; sinus congestion	R	NA	N/A
	18	ACP mucocele	14–26	N/A	VD	R	NA	N/A
Xia et al. 2018 [20]	25	PRL-PA	24	24	VD; H	R	38	cs
Yamaguchi et al. 2016 [19]	35	PRL-PA	36	36	VD; ROP	R	37	cs
Galvão et al. 2017 [18]	30	PRL-PA (PAp)	28	28	consciousness loss; VD; H	R	N/A	N/A
	N/A	PRL-PA (PAp)	25	25	VD; H	R	N/A	N/A
Tandon et al. 2014 [17]	27	PRL-PA (PAp)	19	19	VD; H; nausea	acute symptom worsening at 36 GW	37	cs
Verheecke et al. 2014 [16]	34	CS-M	31	31	VD	R	35	cs
	34	CS-M	27	27	VD; H; nausea	R	33	cs
Moscovici et al. 2014 [1]	39	TS-M	17	15	VD	Pre-eclampsia	35	cs
	34	SpO en plaque M	21	21	VD	Grand mal seizure	41	vl
	33	TS-M	28	27	VD	R	37	cs
	34	ACP-M	29	27	VD	Metabolic acidosis	31	cs
	42	ACP-M	24 (diagnosis after delivery)	24	VD; cognitive impairment	R	39	vl
	35	ACP-M	34	28	VD	R	38	vl
	34	ACP-M	35	30	CN III palsy	R	36	cs
	35	TS-M	32	17	VD	R	38	vl
	40	TS-M	34	24	VD	R	36	cs
	36	TS/ACP-M	32	18	VD	R	39	cs
	28	ACP-M	Delivery (diagnosis after delivery)	38 (delivery)	VD	R	38	vl
Chegour et al. 2014 [15]	19	PRL-PA (PAp)	19	19	VD; H	R	N/A	N/A
Shitara et al. 2012 [14]	29	TS-M	17	15	VD	R	> 36	N/A
Kita et al. 2012 [13]	26	NFPA (PAp)	26	26	VD; H	R	40	vl
Nossek et al. 2011 [12]	29	PA	33	N/A	VD	Early contractions	35	cs
	36	TS-M	29	N/A	VD; CSF leak	R	38	cs
	35	TS-M	29	N/A	VD	R	39	cs
	34	PA	25	N/A	VD	R	40	vl
	38	TS-M	34	N/A	VD	R	36	cs
	32	Petro-clival M	27	N/A	VD; V2-V3 disesthesia	3^rd^ nerve palsy; hemiparesis	34	cs
Lynch et al. 2011 [11]	29	NFPA (PAp)	40	40	blindness	R	40	cs
Iuliano et al. 2011 [10]	28	NFPA (PAp)	29	29	VD; photophobia; ROP	R	39	N/A
	35	NFPA (PAp)	31	33	diplopia (CN VI palsy); ROP	R	39	N/A
Chacko et al. 2010 [9]	27	TS-M	26	24	VD; H; nausea; vomiting	R	30	cs
Ebner et al. 2008 [8]	31	TS-M	31	25	VD	R	34	cs
Abid et al. 2008 [7]	25	PRL-PA	27	27	VD; H	R	39	vl
Atmaca et al. 2006 [6]	33	GH-PA	29	26	VD; acromegaly features	sudden loss of vision and severe headaches at 33 WG	33	cs

*ACP* anterior clinoid process, *CN* cranial nerve, *cs* caesarian section, *CSF* cerebrospinal fluid, *CS-M* Cavernous Sinus Meningioma, *H* headache, *M* meningioma, *N/A* not available, *NFPA* non functioning pituitary adenoma, *PA* pituitary adenoma, *PAp* pituitary apoplexy, *R* regular, *ROP* retro-ortbital pain, *SpO* spheno-orbital, *TS-M* Tuberculum Sellae Meningioma, *VD* visual disturbances, *vl* vaginal labor

^**a**^At symptoms presentation

Meningiomas (25 cases; 50%) were the most common lesions, followed by pituitary adenomas (22 cases, 44%: 11 PRL-, 3 GH-secreting, and 8 non-functioning), 12 (54.5.%) of which presenting with apoplexy. A case of adenoidocystic carcinoma, a pituitary abscess and a mucocele were reported (Table 3).

Pregnancy was uneventful in the great majority of the cases. Complications jeopardizing the mother and the fetus health and life consisted of 2 cases of pre-eclampsia, 1 of grand mal seizures and 1 of metabolic acidosis. Two abortions were also reported. Mean gestational week at delivery was 36.8 ± 2.6 (30–33: n = 5; 34–37: n = 18; 35–41: n = 19; data available for 42 patients). Delivery was by caesarian section in 62.5% of the cases and by vaginal labor in 37.5% (data available for 40 patients) (Table 3). The newborns were healthy; 3 presented with low Apgar score and in 1 with low birth weight.

Tumor exeresis was performed in 46 (92%) cases, of which during pregnancy 27 (58.7%), and after delivery in 15 (32.6%), with similar results in terms of symptoms improvement/recovery, pregnancy course and outcome. Nine patients also received radiation therapy and 3 medical therapy. Three prolactinomas were treated with dopamine agonists during pregnancy with rapid symptoms improvement/resolution. Finally, one patient was not treated (Table 4).

**Table 4:** Results of literature review: type of treatment and outcome

Author, year	Gestational age at *treatment* (week)	Type of treatment	Surgical approach	Additional treatment	Post-treatment outcome	Pregnancy outcome
Zhong et al. 2019 [25]	32	Surgery	EEA	Supraorbital keyhole	Improvement	DHB
	22	Surgery	N/A	None	Improvement	DHB
	18	Surgery	N/A	None	Improvement	abortion
	32	Surgery	N/A	None	Improvement	DHB
Bachmeier et al. 2019 [21]	PP	Surgery	EEA	None	Recovery	DHB
Jemel et al. 2019 [24]	PP	Medical therapy	–	None	Improvement	DHB
	22	Surgery	mTS	None	Improvement	DHB
	24	Surgery	EEA	None	Improvement	DHB
Ennaifer et al. 2018 [22]	36	Medical therapy	–	None	Improvement	DHB
Karaca et al. 2018 [3]	PP (24)	Surgery	N/A	None	Improvement	N/A
	11	Surgery	N/A	None	Improvement	DHB
	9	Surgery	craniotomy	RTx	Improvement	abortion
Priddy et al. 2018 [21]]	> 26	Surgery	EEA	Surgery (FTOZ); RTx	Worsening after 1^st^ surgery; subsequent improvement	N/A
	PP (1)	Surgery	EEA	RTx	Recovery	DHB
	PP (< 18)	Surgery	N/A	None	Stability; metastasis 6 years later	N/A
	PP (> 18)	Surgery	N/A	None	Recovery	DHB
Xia et al. 2018 [20]	24	Surgery	EEA	None	Improvement	DHB
Yamaguchi et al. 2016 [19]	36	Surgery	TS	None	Recovery	DHB
Galvão et al. 2017 [18]	–	None	–	None	Recovery	DHB
	2nd trimester	Surgery	TS	None	Hypopituitarism; symptoms recovery	DHB
Tandon et al. 2014 [17]	36	Surgery	EEA	None	Recovery	DHB
Verheecke et al. 2014 [16]	PP	Surgery	N/A	RTx	Recovery	DHB
	PP	Surgery	N/A	RTx	Recovery	DHB
Moscovici et al. 2014 [1]	20	Surgery	N/A	RTx	Recovery	DHB
	28	Surgery	N/A	RTx	Improvement followed by worsening (tumor regrowth)	DHB
	30	Surgery	N/A	None	Recovery	DHB
	31	Surgery	N/A	None	Recovery	DHB
	PP (7)	Surgery	N/A	None	Recovery	DHB
	PP (12)	Surgery	N/A	None	Recovery	DHB
	PP (11)	Surgery	N/A	RTx	Recovery	DHB
	PP (3)	Surgery	N/A	None	Recovery	DHB
	PP (8)	Surgery	N/A	None	Recovery	DHB
	PP (56)	Surgery	N/A	None	Recovery	DHB
	PP (6)	Surgery	N/A	None	Recovery	DHB
Chegour et al. 2014 [15]	19	Medical therapy	–	–	Recovery	N/A
Shitara et al. 2012 [14]	19	Surgery	Pt	None	Recovery	DHB
Kita et al. 2012 [13]	27	Surgery	EEA	None	Recovery	DHB
Nossek et al. 2011 [12]	33	Surgery	TS	None	Recpvery	Low Apgar score
	31	Surgery	craniotomy	None	Recovery	DHB
	29	Surgery	craniotomy	None	Recovery	Low Apgar score
	31	Surgery	TS	None	Recovery	DHB
	PP (2 days)	Surgery	craniotomy	None	Recovery	DHB
	PP (10 days)	Surgery	craniotomy	None	Recovery	Low Apgar score: low birth weight
Lynch et al. 2011 [11]	PP	Surgery	N/A	None	Partial recovery	N/A
Iuliano et al. 2011 [10]	30	Surgery	TS	None	Recovery	DHB
	33	Surgery	TS	None	Recovery	DHB
Chacko et al. 2010 [9]	PP (40)	Surgery	N/A	None	Recovery	N/A
Ebner et al. 2008 [8]	PP (1)	Surgery	Pt	None	Recovery	DHB
Abid et al. 2008 [7]	27	Surgery	TS	B; L	Recovery	DHB
Atmaca et al. 2006 [6]	33	Surgery	EEA	RTx; SSA	Hypopituitarism	DHB

*B* bromocriptine, *DHB* delivery of a healthy baby, *EEA* endoscopic endonasal approach, *FT* fronto-temporal, *FTOZ* fronto-orbito-zygomatic, *L* Lisuride hydrogen, *mTS* microscopic trans-sphenoidal, *N/A* not available, *PP* postpartum, *Pt* pterional, *RTx* radiotherapy, *SSA* somatostatin analogues, *TS* transcranial

## Discussion

SPTs very rarely become symptomatic during pregnancy, but they can threaten both the mother and the fetus health. Both diagnosis and treatment are very challenging and have to be patient-tailored, balancing the potential for cure and for harm. We reported our experience and the results of a systematic literature review on the management of SPTs.

In line with literature, in our series symptoms mainly occurred in the third trimester, likely because of the progressive tumor growth during pregnancy, as well as for possible intratumor bleeding, favored by the hyperdynamic circulation in the last part of gestation, and pituitary cell hyperplasia driven by placenta hormonal secretion, responsible for mass effect on surrounding structures [1–3, 10].

In literature, meningiomas resulted the most common lesions, clinically presenting during pregnancy, followed by prolactinomas. This could be due to the greater propensity of these tumors to increase in size in response to the estrogen progestin drive, and, thus, become symptomatic during gestation, or to their higher prevalence of these neoplasms in the population.[1]. In particular, pituitary adenomas represent about 15% of intracranial tumors (their prevalence has significantly increased in recent decades and is currently estimated of 115 cases/100,000 in the general population), and prolactinomas account for up to 60% of all pituitary adenomas (up to 75% in women), typically affecting women (female:male ratio of 20:1 for microprolactinoma) in reproductive age [3, 33]. If left untreated, the estimated risk of significant tumor enlargement during pregnancy is reported as 26% for macro- and 1.4% for micro-prolactinomas [2, 34].

Diagnosis of a SPT in pregnancy is typically suggested by the presence of mass-effect symptoms and supported by assessment of hypothalamic-pituitary function and MRI, performed without contrast medium. It has been reported that physiologic functional and imaging alterations associated with pregnancy, together with the need of preventing the mother and the fetus from potentially harmful examinations, strongly influenced the definition of the diagnostic approach and the result interpretation [1–3, 5]. The clinical evaluation represented, even more than for the general population, the main stay of the patient follow-up, to guide tumor management.

At this purpose, it worth to be mentioned that surgery represent the first choice in the treatment of SPTs—except for prolactinomas, for which dopamine agonists are privileged—to achieve complete exeresis (whenever possible) or debulking (with the resolution of compressive symptoms) as soon as possible [33]. However, the management of SPTs in pregnancy is still debated.

The main elements to be considered in deciding whether or not attempting surgery are the mother conditions (i.e. symptoms type and progression), the fetus gestational age and vital parameters, and the predicted outcome of the procedure, since neurosurgical procedures during pregnancy can compromise blood flow to the fetus and induce premature labor [12, 20, 21].

Pregnancy is characterized by immunological modifications, hypercoagulability, fluid shifts and increased intra-abdominal pressure that significantly increase the maternal and fetal anesthesiological and surgical risk in the intra- and peri-operative time [12]. Therefore, although experience is limited, wait and see approach, as well as medical treatment have to be likewise considered because of several reasons [1–3, 5]. First, lymphocytic hypophysitis should be considered in differential diagnosis with SPTs. Indeed, although this is rare condition, it is typically associated to late pregnancy/post-partum. It can occur in the 2nd and 3rd trimester of pregnancy, especially in patients with other autoimmune disorders and presenting with diabetes insipidus and hypopituitarism associated with mass effect symptoms. Moreover, most of clinical, radiological and biochemical features overlap between hypophysitis and other SPTs, but the former has, often, a spontaneous resolution, overall supporting the wait and see approach in patients with mild to moderate symptom severity and stable conditions [35]. Second, ACTH-secreting tumors are quite rare but associated with high rate of complications for the mother (i.e. hypertension or preeclampsia, diabetes, fractures; more rarely, cardiac failure, psychiatric disorders, infection and maternal death) and the fetus (i.e. prematurity, intrauterine growth retardation, and less prevalently stillbirth, spontaneous abortion, intrauterine death, and hypoadrenalism) [34, 36, 37]. Treatment can be aimed at controlling comorbidities, especially in milder cases discovered late in pregnancy. Anticortisolic drugs can be considered while waiting for pituitary surgery, ideally performed in the 2nd-3rd trimester. To date, very few cases of Cushing’s disease have been treated with metyrapone and cabergoline during pregnancy. Other drugs should be avoided for the potential teratogenicity and/or lack of information [34, 36]. Acromegaly does not seem to significantly increase the risk of maternal diabetes mellitus, miscarriage, preterm delivery, nor fetal low birth weight, macrosomia or congenital abnormalities if left untreated during pregnancy. Therefore, surgery should be considered in case of pituitary apoplexy or visual loss. Somatostatin analogues and pegvisomant appear safe [3, 34, 36].

**Fig. 3:**
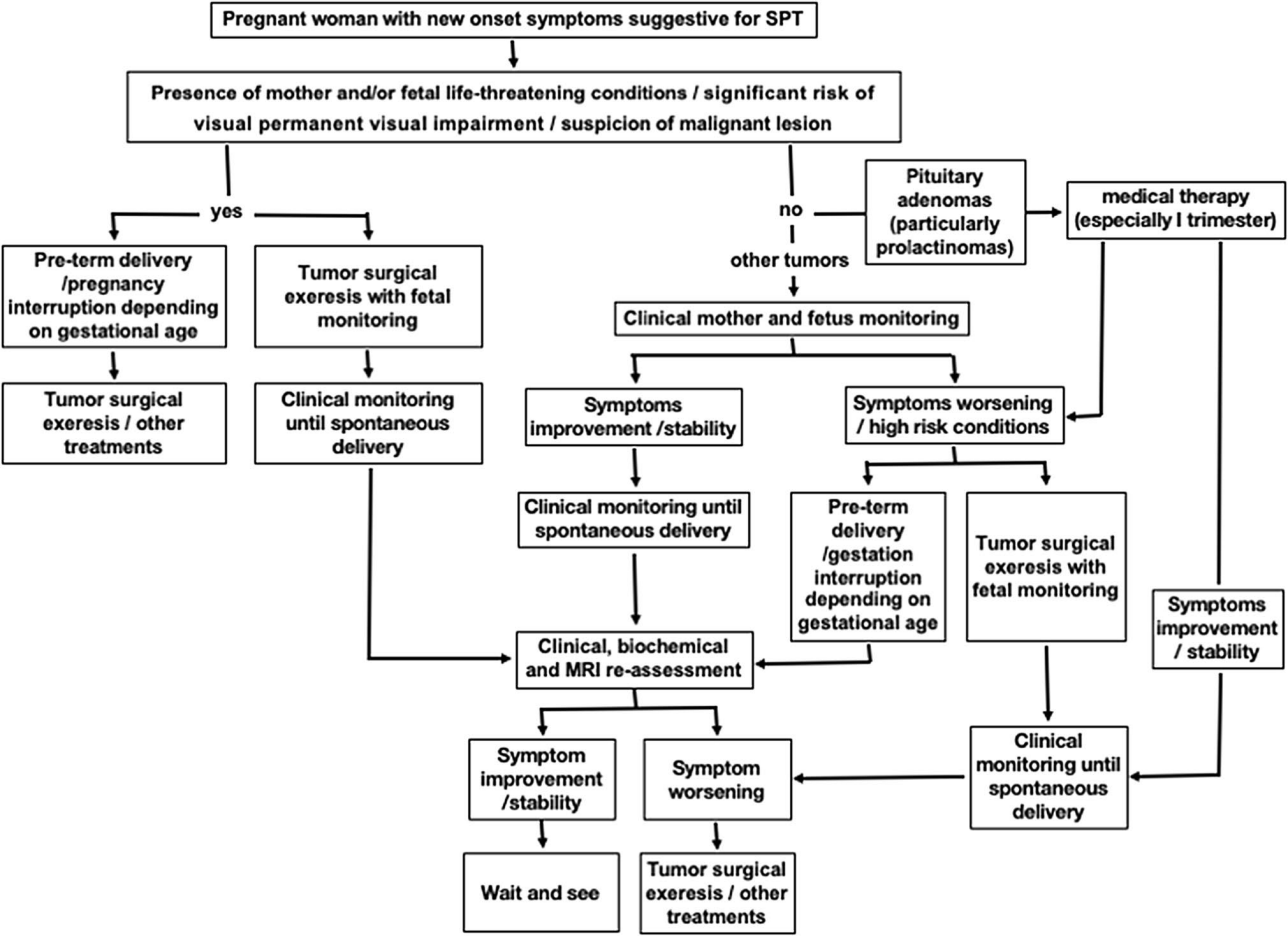
Suggested algorithm for the management of sellar/parasellar tumors (SPTs) becoming symptomatic during pregnancy

Considering all these aspects, together with personal experience and literature review, we propose an algorithm for the management of SPTs becoming symptomatic during pregnancy (Fig. 3). In case of onset of peculiar clinical symptoms or signs highly suggestive for SPT (as for instance visual deficits, severe headache, especially if resistant to pain killer, dizziness, nausea, vomiting, papilledema nystagmus, diplopia, cranial nerve palsies or asthenia), we recommend proceeding with biochemical evaluation, followed by MRI without contrast medium, independently from gestational age. Indeed, even at the first trimester, the potential risks associated with MRI have not been proven to be sufficiently high to justify the serious complications potential associated with the evolution of a symptomatic SPT. The appropriate timing at which performing clinical examination and MRI after symptom onset or patient evaluation cannot be defined a priori, since the severity of symptoms (this the acute or sub-acute presentation) and the evolution of each case are unpredictable. A conservative strategy with careful and close clinical monitoring of mother symptoms (i.e. neurological and ophthalmological evaluation, including visual field test, every 7–10 days or in case of significant subjective changes reported by the patients) and fetal status is suggested in the absence of life-threatening conditions or significant risk of permanent visual impairment and radiological features suspicious for malignant lesions. When clinical conditions are stable or slowly progressive, treatment, especially surgery, should be postponed after delivery. After gestational week 33, delivery can be planned, eventually administering corticosteroids to accelerate fetal lung maturation. Pituitary function, neurological and visual examination, and MRI with contrast medium are recommended in the first time after delivery to re-define surgical indications.

In case of life-threatening maternal or fetal conditions, gestation interruption/induced cesarean delivery (depending on gestational age), followed by prompt surgical intervention has to be considered. Alternatively, if tumor resection could resolve the critical state, as in case of hydrocephalus, intracranial hypertension or optic nerve compression, surgery during pregnancy with fetal intra-operative monitoring can be performed [25].

Despite intrinsic limitations associated with hormonal evaluations in pregnancy, it is extremely important to identify and characterize pituitary adenomas for the possibility of medical treatment, especially for prolactinomas, for the proven efficacy of dopamine agonists to shrink the volume while controlling hormone secretion, waiting for the appropriate timing/in substitution of surgery, therefore for these cases surgery should be considered strictly limiting to those patients considered extremely high risk of life-threatening complications, or already treated with dopamine agonists presenting with rapid and significant symptom worsening [26] (Fig. 3).

No specific guidelines exist to define the optimal delivery method. Spontaneous vaginal labor, for the uterine contractions and the extreme Valsalva’s maneuver, is associated with an important increase of intracranial pressure, potentially dangerous in patients with a SPT or in the early post-operative time for the risk of CSF leak after a transsphenoidal surgery [21].

Main strength of the study are the systematic literature review and the suggested practical algorithm for the management of SPTs becoming symptomatic during pregnancy, while the retrospective design and the inclusion of different types of tumors, necessary to reach a reasonable number of cases for data analysis, represent not negligible study limitations.

## Conclusions

SPTs becoming symptomatic during pregnancy should be referred to Pituitary Centers, since their management is extremely challenging and requires a dedicated trained team. A practical algorithm to guide physicians dealing with this condition is proposed, overall privileging wait and see approach with careful patient and fetus monitoring, while reserving surgery during pregnancy to clinically unstable patients, with life-threatening/fast worsening situations or suspected malignant lesions.

## Data Availability

Study results have been presented in part as an oral communication at the 68th Congress of the Italian Society of Neurosurgery, Rome, 16-18th September 2019. Two of the cases reported in this manuscript had also been previously published as case reports (see references 31 and 32).
